# Is subjective sleep evaluation a good predictor for obstructive sleep apnea?

**DOI:** 10.6061/clinics/2018/e355

**Published:** 2018-06-23

**Authors:** Cárita de Moura Laranjeira, Eline Rozária Ferreira Barbosa, Marcelo Fouad Rabahi

**Affiliations:** I Programa de Pos-Graduacao, Universidade Federal de Goias, Goiania, GO, BR; II CLARE – Clinica do Aparelho Respiratorio e Medicina do Sono, Goiania, GO, BR

**Keywords:** Sleep Apnea, Obstructive, Perception, Physiopathology, Polysomnography, Questionnaires

## Abstract

**OBJECTIVE:**

To compare subjective sleep evaluation obtained using four questionnaires with polysomnography results for individuals with and without obstructive sleep apnea.

**METHODS:**

Observational and analytical study in which individuals underwent polysomnography were studied retrospectively to investigate sleep disorders. We compared subjective data from a research database used to predict obstructive sleep apnea based on the STOP-BANG questionnaire, evaluation of excessive daytime sleepiness (Epworth Sleepiness Scale), sleep quality questionnaire (Mini Sleep Questionnaire) and Post-Sleep Data Collection Instrument with the self-reported total sleep time and sleep-onset latency for subjects with and without obstructive sleep apnea.

**RESULTS:**

The STOP-BANG questionnaire was a good predictor for the diagnosis of obstructive sleep apnea. However, the other instruments did not show a significant difference between healthy and sick individuals. Patients' perceptions of their sleep onset time were significantly lower than the polysomnographic data, but this difference remained for both subjects with and without obstructive sleep apnea. No difference was found between the subjective duration of sleep and the total sleep time assessed by polysomnography in either the healthy subjects or the patients.

**CONCLUSION:**

Except for the STOP-BANG questionnaire, subjective evaluation of sleepiness, sleep quality, perception of onset, and total sleep time are not important parameters for the diagnosis of obstructive sleep apnea, which reinforces the need for an active search for better management of these patients.

## INTRODUCTION

Sleep is a complex behavioral state involving a reversible reduction of perception and relative unresponsiveness to the environment 1. Perception of the amount of sleeping hours and sleep quality are subjective information and may not agree with objective measures. Therefore, diagnosing sleep disorders based only on symptoms reported by patients is a great challenge 2,3.

A mismatch between subjective data (patient complaints) and objective (polysomnographic) measurements may be related to fragmented sleep 2 in both insomnia and apneic patients. Polysomnography (PSG) is the gold standard for the diagnosis of sleep-disordered breathing 3. PSG is recommended for the diagnosis of obstructive sleep apnea after clinical evaluation 4 and in patients with potential respiratory muscle weakness due to neuromuscular diseases, hypoventilation (sleep or wakefulness), chronic use of opioids and a history of stroke or severe insomnia 5.

A poor perception of sleep is defined as a total sleep time (TST) reported by the subject that is less than 80% of the total sleep time measured by PSG 6-8. Therefore, an individual with a sleep disorder may not perceive events that indicate the presence of morbidity. Identification of sleep perception patterns can help differentiate between normal individuals and those with altered sleep and consequently enable an early diagnosis, treatment and greater compliance in individuals with altered sleep.

Obstructive sleep apnea (OSA) is a disease associated with several cardiovascular 9,10, respiratory 11,12, metabolic 13,14 and even oncologic problems 15-17. The prevalence of OSA varies according to the criteria used, with a range from 10% to 49.7% in men and 3% to 23.4% in women (AHI≥15 events/h) 18-21. Several studies have demonstrated high rates of underdiagnosis 22-24. Complaints related to OSA are often nonspecific, and the perception of sleep disturbance can be a warning factor for a better diagnostic accuracy 25.

Thus, the purpose of this study is to compare subjective sleep evaluation using four questionnaires with polysomnography results in individuals with and without OSA.

## METHODS

This study was an observational and analytical study that utilized PSG to determine the presence of sleep disorders. PSG data from a research database were retrospectively compared to subjective OSA assessment data. The subjective evaluation was performed through four questionnaires [the STOP-BANG questionnaire, Epworth Sleepiness Scale, a sleep quality questionnaire (Mini Sleep Questionnaire - MSQ) and Post-Sleep Data Collection Instrument (PSDCI)] to assess the self-reported total sleep time and sleep-onset latency of subjects with and without OSA.

The study analyzed individuals who underwent PSG from January to December 2015 at the Respiratory Research Center of an outpatient Chest and Sleep Medicine Clinic (CLARE) in Goiânia, Goiás, Brazil. The inclusion criteria were as follows: individuals over 18 years of age who underwent a complete PSG and signed the consent form. The exclusion criteria was individuals with incomplete data in their questionnaires and/or sleep perception forms.

The demographic characteristics collected included gender, age, and the body mass index (BMI). These data were correlated with the apnea and hypopnea index (AHI) of each patient obtained through PSG. The following three validated questionnaires for Portuguese were used for the subjective evaluation of the patient's perception: 1. Risk assessment for OSA: STOP-BANG (≤2: low risk; 3-4: intermediate risk; and ≥5: high risk) 26,27; 2. Evaluation of daytime sleepiness: Epworth Sleepiness Scale (≤10: normal; 11-15 drowsiness; and ≥16: severe drowsiness 28,29; and 3. Sleep quality assessment: Mini-sleep Questionnaire - MSQ (10-24: good sleep, 25-27: slightly altered sleep, 28-30: moderately altered sleep, and >30: heavily altered sleep) 30,31. In addition, the PSDCI was used as a subjective assessment complement immediately after the PSG. This instrument evaluated the degree of sleep discomfort in the sleep lab, the perceived time to sleep onset, the self-reported total amount of sleep, and how many times the individual reported waking during the night of the examination. Sleep perception was defined as the percentage of the ratio between the perceived TST by the subject and the TST measured by the PSG 6. Poor sleep perception was defined as a TST reported by the subject that was less than 80% of the TST measured by PSG 6-8.

PSG was performed with an ALICE-5 device (Philips Respironics, PA) in a sleep laboratory to evaluate multiple neurophysiological variables during the night of recording, including an electroencephalogram, submental and leg electromyogram (bilateral), electrocardiogram, nasal flow, respiratory effort by thoracic and abdominal straps, snoring sensor and pulseoximetry (oxyhemoglobin saturation). The exams were scored by medical specialists in Sleep Medicine. The presence of an AHI≥5 events/hour was considered for the diagnosis of OSA according to the current criteria from the American Academy of Sleep Medicine (AASM) 32.

The present study was approved by the Research Ethics Committee of the Federal University of Goiás under number CAAE: 56008316.7.0000.5083.

### Statistical Analysis

A t-test was used to define the sample size. We chose an α (two-tailed)=0.01 and power=0.95 33. The sample was calculated to be able to detect a difference of 20% or more in the perception of sleep between the objective and subjective evaluation of each individual and to differentiate normal from abnormal perceptions. The sleep perception was defined as the ratio between the sleep duration (self-reported) and TST (polysomnographic data) 6. A proportion less than 80% was considered an altered sleep perception. In addition, a previous study reported a mean and standard deviation of sleep perception of 0.82±0.20 7.

The data were analyzed using the statistical package SPSS (Statistical Package for Social Science) version 23. The level of significance was set at 5% (*p*<0.05). The sample was characterized based on the absolute and relative frequencies for categorical variables (Chi-square test - χ^2^) and means with standard deviations for continuous variables (Kruskal-Wallis test). The normality of the data was verified by applying the Shapiro-Wilk test. If the normality of the data was not verified, non-parametric statistics were adopted. Comparisons were performed between subjective and objective data for the apneic and non-apneic participants (box-plot graphs), with *p* values obtained through Wilcoxon's non-parametric test.

The dispersion curves (Figure 2) compared the polysomnographic (x-axis) measurements with the following self-reported questions using Spearman's correlation: (A) how many times the participant reported having awakened, (B) the sleep duration and (C) the time elapsed for sleep onset for the total sample.

For Spearman's correlation between the subjective (“perception”) and objective (polysomnographic) data, positive values demonstrate agreement between the reported and measured findings (if *p*<0.05), and negative values demonstrate inverse correlations.

## RESULTS

**Figure 1:**
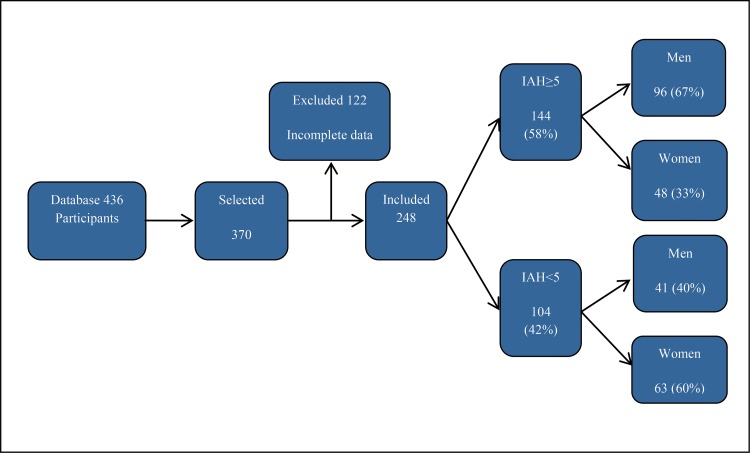
Selection criteria and sample distribution for the Apnea Hypopnea Index (AHI) and gender.

**Figure 2:**
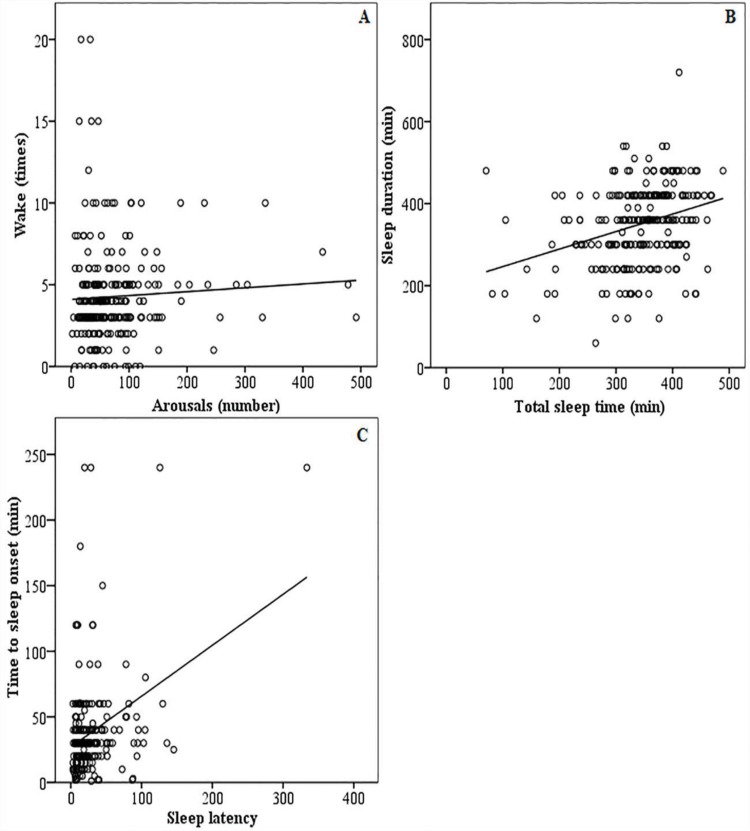
(A, B, and C). Dispersion curves comparing the self-reported (y-axis) and polysomnographic (x-axis) measurements of the 248 participants using Spearman's correlation. The straight line demonstrates a perfect correlation between the subjective and objective measures. Figure 2A shows the number of awakenings perceived in relation to arousals. Figure 2B shows the correlation between the perceived sleep time and the total sleep time (TTS). Figure 2C shows the discrepancy between the time perceived for the beginning of sleep and the sleep latency.

A total of 436 individuals who underwent PSG were selected from January to December 2015. After applying the inclusion and exclusion criteria, we obtained 248 participants, as shown in Figure 1.

The evaluated subjects included 137 men (55.2%) and 111 women (44.8%). The mean age was 40±12.7 years for participants with an AHI<5 and 46±12.6 years for an AHI≥5 events/hour (*p*=0.05). The frequency of OSA was higher in men (70.1%) than in women (43.2%), (*p*<0.001, Chi-square test).

Participants presented with the following characteristics: normal BMI, 32 (12.9%); overweight, 61 (24.6%) and obesity, 155 (62.5%). In the obese category, 46 (29.7%) participants were classified with obesity class I, 45 (29.0%) with class II and 64 (41.3%) with morbid obesity. The BMIs of the apneic patients (36.8±8.3) were significantly higher than those of the participants with a normal AHI (31±20.5), (*p*<0.001, Chi-square test).

The presence of sleep apnea was confirmed (Table 1) in 144 (58.0%) of the sample, and no significant difference was found between the adults and the elderly (*p*=0.05). The frequency of OSA was greater among the males than among the females (2:1, *p*<0.001).

The STOP-BANG questionnaire was a good predictor for the diagnosis of OSA (*p*<0.001), but the other instruments did not show a significant difference between the healthy and sick individuals. The STOP-BANG questionnaire showed a correlation with OSA in both women and men (*p*<0.001 and *p*=0.01, respectively). The Epworth Sleepiness Scale (Table 1) did not correlate with the disease in this sample (*p*=0.54). Conversely, the MSQ showed a correlation in the total sample between poor sleep and OSA (*p*=0.04).

**Figure 3:**
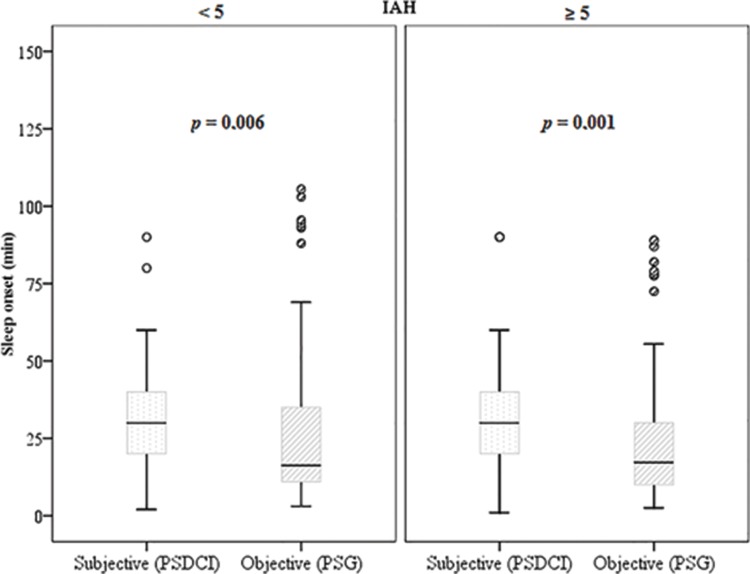
Boxplot comparing the sleep onset time (subjective data, *PSDCI)* and sleep latency (PSG) in minutes separated between apneic (AHI>5) and non-apneic patients using Wilcoxon's non-parametric test. PSDCI: Post-Sleep Data Collection Instrument. PSG: polysomnography.

Thus, a positive correlation was found between the onset of sleep and sleep latency in women (*p*<0.001) and adults (*p*=0.001). Patients with obstructive sleep apnea had a negative correlation between sleep onset and latency (ρ=-0.29 and *p*<0.001). Wilcoxon's non-parametric test (Figure 3) showed that the apneic patients perceived sleep onset as significantly greater than the objective data (overestimating latency), (*p*<0.001).

A positive correlation in the amount of perceived sleep time in relation to the total sleep time was found in all groups (*p*<0.001 and ρ=0.31). However, Wilcoxon's non-parametric test (Figure 4) showed that this difference was not significant in the non-apneic patients and in the participants with an AHI>5 (*p*=0.57 and *p*=0.26, respectively) when the sleep perception was compared with the objective data.

PSG showed a good correlation (*p*<0.001) with the presence of OSA in all categories (both genres and age groups). Notably, the number of awakenings perceived by the patient (which in the sample ranged from 0 to 20) did not correlate by definition with the arousal index measured according to the AASM criteria 32. The relationship of arousals with the awakening perception was not significant.

## DISCUSSION

The main finding of this study is that subjective instruments for the detection of sleep disorders do not differentiate between sick and healthy individuals to predict OSA, with the exception of STOP-BANG. Thus, the perception of sleep onset and its total duration did not help differentiate between the presence and absence of OSA compared to the polysomnographic data.

This finding is surprising, since some studies have reported a discrepancy between the perception of TTS and the objective data from these patients obtained with PSG 2,37-39. Thus, the importance of the active search for OSA-related symptoms is emphasized, and the use of only perception of sleep reported by the patient is not recommended. For this purpose, we used risk assessment questionnaires for the OSA risk, such as the STOP-BANG questionnaire, which was shown to be a good predictor compared to PSG, as shown in this study. The agreement of sleep perception between apneic and non-apneic individuals was also found previously, which reinforced that sleep perception was not a good parameter for assessment of the presence of OSA 7.

Perception studies are more frequent with insomnia, since the discrepancy between perceived sleep and measured sleep in these patients has been widely described 2,6. The literature concerning the analysis of respiratory sleep disorders is controversial, since higher sleep efficiency has been found in the group with isolated OSA (without associated insomnia) despite demonstrating the worst AHI and a high index of arousal 8. Other authors reported data similar to the data obtained for insomniacs, with an overestimation of sleep latency and an underestimated TST 25,26. The present study presented a miscellany of these reports, with no difference in the TST in apneic patients compared to the polysomnographic data 2 and an overestimation of the sleep latency, which was significant in both the apneic and non-apneic patients.

Conversely, the association between OSA and complaints of insomnia was high, with a range from 42% to 51.8% 35-37. This finding also led to an alteration in sleep perception (worse in insomniacs, intermediate in insomnia comorbid to OSA, and in a lower proportion in patients with isolated OSA), thereby establishing a continuum of poor perception 2,38.

Some hypotheses have been reported for nocturnal sleep apnea amnesia, since intermittent hypoxia itself is a factor capable of affecting memory (spatial and working), even in young adults with no comorbidities 38, in addition to OSA disrupting deep sleep and the reduction of REM sleep. Therefore, by not completing a sleep cycle, the perception can be altered to the point where the sleep period is considered as wakefulness (when the patient is awakened during REM sleep). In addition, a quality sleep report did not correlate with the OSA severity 7, which was consistent with our results in which the OSA severity did not correlate with a poorer sleep perception.

Arousals (duration of less than 15 seconds) are not perceived as interruptions of sleep by the cerebral cortex, and therefore there is no correlation with the awakenings reported by the patient. However, a high arousal index may be indicative of the presence and severity of apnea and other possible sleep disturbances. In an experimental model, healthy participants were subjected to moderate respiratory overload, and the increase of arousals correlated with an increase in the perception of the sleep onset time 39. In the present study, the high frequency of arousals correlated with the presence of OSA but did not affect the perception of sleep.

The sample includes 58.0% apneic patients, which can be attributed to the selection of patients referred to the clinic by physicians to perform PSG. The high prevalence of OSA is also expected due to the considerable frequency of obese people in this population (62.5%). The prevalence of apnea in obese individuals is increased, reaching 85.7% in obese class III patients who are awaiting bariatric surgery 40.

The limitations of this study included the use of medications, diagnosis of other associated sleep disorders (mainly insomnia) and possible comorbidities that were not evaluated. The architecture of sleep was not studied to verify differences between groups.

The strength of this study is that it demonstrates a concern about the usefulness of subjective evaluation for respiratory sleep disturbances. Except for the use of the STOP-BANG questionnaire, subjective evaluation of daytime sleepiness, sleep quality, and perception of onset and total sleep time are not important parameters for the diagnosis of obstructive sleep apnea, reinforcing the need for an active search and objective measurements for better sleep management of these patients.

### Conflicts of Interest

MFR: research grants and/or speaker's honoraria from AstraZeneca, Novartis and Boehringer. Financing: none.

## AUTHOR CONTRIBUTIONS

Laranjeira CM was responsible for delineation and data collection. Barbosa ER was responsible for manuscript writing. Rabahi MF was responsible for the analysis of results and supervision.

## Figures and Tables

**Figure:**
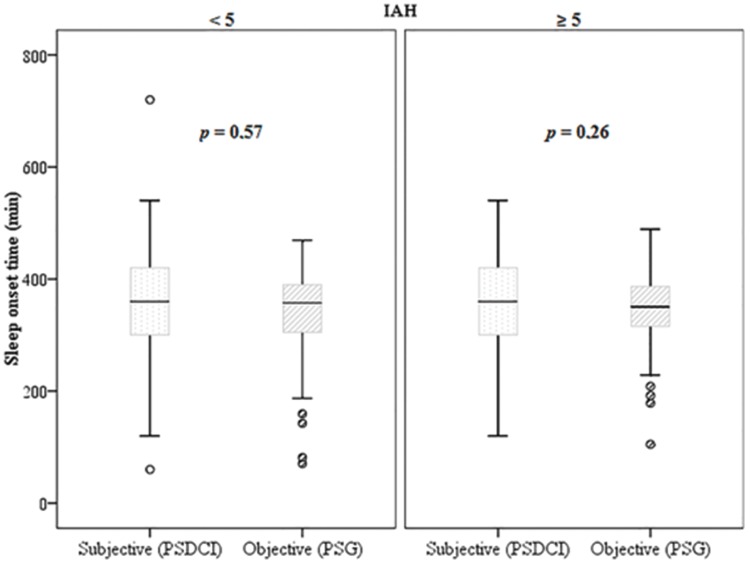
Boxplot comparing the perception of how much the individual slept (PSDCI) and the total sleep time (PSG) separated between apneic (AHI>5) and non-apneic patients using the non-parametric Wilcoxon test. PSDCI: Post-Sleep Data Collection Instrument. PSG: polysomnography.

**Table 1:** Characterization of sleep evaluation regarding the presence of OSA in 248 individuals subjected to PSG.

Evaluation forms	No OSA n (%) 104 (42%)	With OSA n (%) 144 (58%)	*p*
**SUBJECTIVE**			
***Risk of OSA (STOP-BANG)***			*p*<0.001+
Low	36(14.5%)	15(6.0%)	
Intermediate	49(19.8%)	67(27.0%)	
High	19(7.7%)	62(25.0%)	
***Evaluation of daytime sleepiness (ESS)***			*p*=0.54+
<10 (normal)	55(22.1%)	82(33.1%)	
10-16 (moderate)	32(12.9%)	43(17.3%)	
>16 (severe)	17(6.9%)	19(7.7%)	
***Quality of sleep evaluation (MSQ)***			*p*=0.04+
Good	11(4.4%)	16(6.5%)	
Slightly altered	04(1.6%)	14(5.7%)	
Moderately altered	11(4.4%)	21(8.5%)	
Poor	78(31.4%)	93(37.5%)	
***Evaluation of sleep perception (PSDCI)***			
Sleep onset (min±SD)	39.9±14.9	35.5±40.3	*p*=0.06+
Sleep duration (min±SD)	350.6±16.0	362.2±105.1	*p*=0.93+
How many times did you wake up (n)	4.2±16.4	4.3±2.9	*p*=0.23+
**OBJECTIVE**			
***(PSG)***			
Sleep latency (min±SD)	32.2±80.9	23.4±43.4	*p*=0.11+
Total sleep time (min±SD)	339.6±75.0	345.9±75.5	*p*=0.21+
Arousals (n±SD)	40.0±77.7	96.8±24.4	*p*<0.001+

OSA: obstructive sleep apnea; SD: standard deviation; BMI: body mass index; MSQ: Mini Sleep Questionnaire; PSG: polysomnography; ESS: Epworth Sleepiness Scale; PSDCI: Post-Sleep Data Collection Instrument; + Spearman Correlation.

## References

[1] Carskadon M, Dement W, Kryger M, Roth T, Dement W (2005). Normal human sleep: an overview. Principles and practice of sleep medicine.

[2] Bianchi MT, Williams KL, McKinney S, Ellenbogen JM (2013). The subjective-objective mismatch in sleep perception among those with insomnia and sleep apnea. J Sleep Res.

[3] Practice parameters for the indications for polysomnography and related procedures (1997). Polysomnography Task Force, American Sleep Disorders Association Standards of Practice Committee. Sleep.

[4] Kushida CA, Littner MR, Morgenthaler T, Alessi CA, Bailey D, Coleman J (2005). Practice parameters for the indications for polysomnography and related procedures: an update for 2005. Sleep.

[5] Kapur VK, Auckley DH, Chowdhuri S, Kuhlmann DC, Mehra R, Ramar K (2017). Clinical Practice Guideline for Diagnostic Testing for Adult Obstructive Sleep Apnea: An American Academy of Sleep Medicine Clinical Practice Guideline. J Clin Sleep Med.

[6] Pinto LR, Pinto MC, Goulart LI, Truksinas E, Rossi MV, Morin CM (2009). Sleep perception in insomniacs, sleep-disordered breathing patients, and healthy volunteers - an important biologic parameter of sleep. Sleep Med.

[7] Nam H, Lim JS, Kim JS, Lee KJ, Koo DL, Lee C (2016). Sleep Perception in Obstructive Sleep Apnea: A Study Using Polysomnography and the Multiple Sleep Latency Test. J Clin Neurol.

[8] Choi SJ, Suh S, Ong J, Joo EY (2016). Sleep Misperception in Chronic Insomnia Patients with Obstructive Sleep Apnea Syndrome: Implications for Clinical Assessment. J. Clin Sleep Med.

[9] Peker Y, Carlson J, Hedner J (2006). Increased incidence of coronary artery disease in sleep apnoea: a long-term follow-up. Eur Respir J.

[10] Drager LF, Genta PR, Pedrosa RP, Nerbass FB, Gonzaga CC, Krieger EM (2010). Characteristics and predictors of obstructive sleep apnea in patients with systemic hypertension. Am J Cardiol.

[11] Silva JL (2014). Depression and sleep disorders among patients with Chronic Obstructive Pulmonary Disease [thesis].

[12] Soler X, Liao SY, Marin JM, Lorenzi G, Jen R, Deyoung P (2017). Age, gender, neck circumference, and Epworth sleepiness scale do not predict obstructive sleep apnea (OSA) in moderate to severe chronic obstructive pulmonary disease (COPD): The challenge to predict OSA in advanced COPD. Plos One.

[13] Drager LF, Togeiro SM, Polotsky VY, Lorenzi-Filho G (2013). Obstructive sleep apnea: a cardiometabolic risk in obesity and the metabolic syndrome. J Am Coll Cardiol.

[14] Punjabi NM, Polotsky VY (2005). Disorders of glucose metabolism in sleep apnea. J Appl Physiol.

[15] Nieto FJ, Peppard PE, Young T, Finn L, Hla KM, Farré R (2012). Sleep-disordered breathing and cancer mortality: results from the Wisconsin Sleep Cohort Study. Am J Respir Crit Care Med.

[16] Gozal D, Ham SA, Mokhlesi B (2016). Sleep Apnea and Cancer: Analysis of a Nationwide Population Sample. Sleep.

[17] Campos-Rodríguez F, Martinez-Garcia MA, Martinez M, Duran-Cantolla J, Peãa Mde L, Masdeu MJ (2013). Association between obstructive sleep apnea and cancer incidence in a large multicenter Spanish cohort. Am J Respir Crit Care Med.

[18] Young T, Palta M, Dempsey J, Peppard PE, Nieto FJ, Hla KM (2009). Burden of sleep apnea: rationale, design, and major findings of the Wisconsin Sleep Cohort study. WMJ.

[19] Tufik S, Santos-Silva R, Taddei JA, Bittencourt LR (2010). Obstructive sleep apnea syndrome in the Sao Paulo Epidemiologic Sleep Study. Sleep Med.

[20] Peppard PE, Young T, Barnet JH, Palta M, Hagen EW, Hla KM (2013). Increased prevalence of sleep-disordered breathing in adults. Am J Epidemiol.

[21] Heinzer R, Vat S, Marques-Vidal P, Marti-Soler H, Andries D, Tobback N (2015). Prevalence of sleep-disordered breathing in the general population: the HypnoLaus study. Lancet Respir Med.

[22] Kapur V, Strohl KP, Redline S, Iber C, O’Connor G, Nieto J (2002). Underdiagnosis of sleep apnea syndrome in U.S. communities. Sleep Breath.

[23] Rodrigues AP, Pinto P, Nunes B, Bárbara C (2017). Obstructive Sleep Apnea: Epidemiology and Portuguese patients profile. Rev Port Pneumol.

[24] Lorenzi-Filho G, Genta PR, Drager LF (2017). Are we missing obstructive sleep apnea diagnosis?. Rev Port Pneumol.

[25] Castillo J, Goparaju B, Bianchi MT (2014). Sleep-wake misperception in sleep apnea patients undergoing diagnostic versus titration polysomnography. J Psychosom Res.

[26] Chung F, Subramanyam R, Liao P, Sasaki E, Shapiro C, Sun Y (2012). High STOP-Bang score indicates a high probability of obstructive sleep apnea. Br J Anaesth.

[27] Fonseca LB, Silveira EA, Lima NM, Rabahi MF (2016). Tradução e adaptação transcultural do questionário STOP-BANG para a língua portuguesa falada no Brasil. J Bras Pneumol.

[28] Johns MW (1991). A new method for measuring daytime sleepiness: the Epworth sleepiness scale. Sleep.

[29] Bertolazi A (2008). Tradução, adaptação cultural e validação de dois instrumentos de avaliação do sono: escala de sonolência de Epworth e índice de qualidade do sono de Pittsburgh.

[30] Zomer J, Peled R, Rubin A, Lavie P, Koella WP, Rüther E, Schulz H (1985). Mini-Sleep Questionnaire (MSQ) for screening large populations for EDS complaints. Sleep.

[31] Falavigna A, de Souza Bezerra ML, Teles AR, Kleber FD, Velho MC, da Silva RC (2011). Consistency and reliability of the Brazilian Portuguese version of the Mini-Sleep Questionnaire in undergraduate students. Sleep Breath.

[32] Berry RB, Brooks R, Gamaldo CE, Harding SM, Lloyd RM, Marcus CL (2015). The AASM Manual for the Scoring of Sleep and Associated Events: Rules, Terminology and Technical Specifications, Version 2.2.

[33] Hulley S, Cummings S, Hulley SB, Cummings SR (2007). Estimating sample size and power: applications and examples. Designing clinical research.

[34] McCall MV, Turpin E, Reboussin D, Edinger JD, Haponik EF (1995). Subjective estimates of sleep differ from polysomnographic measurements in obstructive sleep apnea patients. Sleep.

[35] Chung KF (2005). Insomnia subtypes and their relationships to daytime sleepiness in patients with obstructive sleep apnea. Respiration.

[36] Krell SB, Kapur VK (2005). Insomnia complaints in patients evaluated for obstructive sleep apnea. Sleep Breath.

[37] Benetó A, Gomez-Siurana E, Rubio-Sanchez P (2009). Comorbidity between sleep apnea and insomnia. Sleep Med Rev.

[38] Champod AS, Eskes GA, Foster GE, Hanly PJ, Pialoux V, Beaudin AE (2013). Effects of acute intermittent hypoxia on working memory in young healthy adults. Am J Respir Crit Care Med.

[39] Smith S, Trinder J (2000). The effect of arousals during sleep onset on estimates of sleep onset latency. J Sleep Res.

[40] Kositanurit W, Muntham D, Udomsawaengsup S, Chirakalwasan N (2018). Prevalence and associated factors of obstructive sleep apnea in morbidly obese patients undergoing bariatric surgery. Sleep Breath.

